# Regenerative effects of human chondrocyte sheets in a xenogeneic transplantation model using immune‐deficient rats

**DOI:** 10.1002/term.3101

**Published:** 2020-07-22

**Authors:** Daichi Takizawa, Masato Sato, Eri Okada, Takumi Takahashi, Miki Maehara, Ayako Tominaga, Yasuyuki Sogo, Eriko Toyoda, Masahiko Watanabe

**Affiliations:** ^1^ Department of Orthopaedic Surgery, Surgical Science Tokai University School of Medicine Isehara Japan; ^2^ Center for Musculoskeletal innovative Research and Advancement (C‐MiRA) Tokai University Graduate School Isehara Japan; ^3^ Department of Orthopaedic Surgery Tokyo Women's Medical University Shinjuku‐ku Japan

**Keywords:** allogeneic, transplantation, articular, cartilage, chondrocytes, preclinical, transplantation, xenogeneic transplantation

## Abstract

Although cell transplantation has attracted much attention in regenerative medicine, animal models continue to be used in translational research to evaluate safety and efficacy because cell sources and transplantation modalities are so diverse. In the present study, we investigated the regenerative effects of human chondrocyte sheets on articular cartilage in a xenogeneic transplantation model using immune‐deficient rats. Osteochondral defects were created in the knee joints of immune‐deficient rats that were treated as Group A, untreated (without transplantation); Group B, transplantation of a layered chondrocyte sheet containing 5.0 × 10^5^ cells (layered chondrocyte sheet transplantation); Group C, transplantation of a synoviocyte sheet containing 5.0 × 10^5^ cells (synoviocyte sheet transplantation); or Group D, transplantation of both a synoviocyte sheet plus a layered chondrocyte sheet, each containing 5.0 × 10^5^ cells (synoviocyte sheet plus layered chondrocyte sheet transplantation). Histological evaluation demonstrated that Group B showed cartilage regeneration with hyaline cartilage and fibrocartilage. In Groups C and D, the defect was filled with fibrous tissue but no hyaline cartilage. Transplanted cells were detected at 4 and 12 weeks after transplantation, but the number of cells had decreased at 12 weeks. Our results indicate that layered chondrocyte sheet transplantation contributes to articular cartilage regeneration; this model proved useful for evaluating these regenerative effects.

## 1 INTRODUCTION

Articular cartilage comprises sparsely distributed chondrocytes in a rich extracellular matrix consisting of type II collagen and proteoglycans. It primarily functions to absorb shock and facilitate smooth articular movements (Buckwalter & Mankin, 1998; Buckwalter, Mankin, & Grodzinsky, 2005). Because articular cartilage is an avascular tissue, it has little potential for self‐repair after injuries or lesions, which often worsen over time, leading to knee osteoarthritis (OA; Brittberg et al., 2016).

Current surgical approaches for small cartilage defect include bone marrow stimulation (Steadman, Rodkey, & Briggs, 2002), osteochondral autograft transfer (Hangody et al., 2004), osteochondral allograft transplantation (Bugbee, Pallante‐Kichura, Görtz, Amiel, & Sah, 2016), and autologous chondrocyte implantation (Brittberg et al., 1994; Saris et al., 2009). Although clinical outcomes suggest some success, those approaches are insufficient for evidence, tissue engineer approaches are emerging as alternatives to current surgical approaches for cartilage repair (Kwon et al., 2019; Makris, Gomoll, Malizos, Hu, & Athanasiou, 2015). Especially using scaffold‐free approach, we produced chondrocyte sheets fabricated on temperature‐responsive cell culture inserts, which allow the transplantation of chondrocytes with their extracellular matrix intact (Kaneshiro et al., 2007; Kokubo et al., 2016; Mitani et al., 2009). Translational models including rats (Takaku et al., 2014; Takatori et al., 2018), rabbits (Ito et al., 2012; Kaneshiro et al., 2006; Tani et al., 2017), and minipigs (Ebihara et al., 2012) were used previously to confirm the safety and efficacy of chondrocyte sheet transplantation. Next, we conducted a clinical study using autologous transplantation of human chondrocyte sheets to treat patients with knee OA, after which we detected no severe adverse events after more than 3 years of follow‐up. In addition, histological analysis of the regenerated cartilage demonstrated that chondrocyte sheet transplantation could lead to regeneration of hyaline cartilage (Sato et al., 2019). Recently, we reported the mode of action of chondrocyte sheets in hyaline cartilage regeneration by transcriptomic and proteomic analyses (Toyoda et al., 2019). We are currently conducting our second clinical study using allogeneic transplantation of chondrocyte sheets fabricated from unwanted surgical tissue derived from patients with polydactyly (Maehara et al., 2017).

To evaluate new human cell sources of chondrocyte sheets, it is preferable to use translational models that can directly evaluate the final product. In other words, xenogeneic orthotopic transplantation models that approximate the in vivo efficacy of clinical transplantation are required. We previously reported the use of an immunosuppressed rabbit xenogeneic transplantation model to directly evaluate human chondrocyte sheets for articular cartilage repair (Takahashi et al., 2018), but the duration for which immunosuppression was effective limited long‐term studies. Ito et al. (2012) and Shimizu et al. (2015) reported conflicting results concerning the effectiveness of chondrocyte sheets and synoviocyte (SY) sheets using allogeneic transplantation in rabbits and rats, respectively. In the present study, we investigated the regenerative effects of human chondrocyte sheets and SY sheets for articular cartilage regeneration in a xenogeneic model using immune‐deficient rats to establish a translational model that can approximate the efficacy of allogeneic transplantation of human chondrocyte sheets.

## 2 MATERIALS AND METHODS

All procedures using animals in this study were performed in accordance with the Guide for the Care and Use of Laboratory Animals (NIH Publication No. 85‐23, revised 2010) published by the National Institutes of Health, USA, and the Guidelines of Tokai University on Animal Use (Authorization No. 173035). Articular chondrocytes and synoviocytes were collected and used after obtaining patients' informed consent and with the approval of our Hospital's Clinical Research Review Committee (Authorization No. 09R‐070).

### 2.1 Fabrication of LC sheets and SY sheets

Articular cartilage and synovial tissues were collected from the unwanted postoperative tissues of six patients (age 71–78 years; average age 74 years) who underwent total knee arthroplasty at Tokai University Hospital. Articular cartilage and synovial tissues were enzymatically digested separately to isolate chondrocytes and synoviocytes, respectively. Layered chondrocyte sheets (LC sheets) and SY sheets were made according to the methods described by Kokubo et al. (2016) and Takahashi et al. (2018), as summarized below. Briefly, articular cartilage or synovial tissues were minced in a petri dish using scissors, and then digested in Dulbecco's modified Eagle's medium‐Nutrient Mixture F‐12 (DMEM/F12; Gibco, Waltham, MA, USA) containing 5 mg/ml collagenase type I (Worthington, NJ, USA), 20% fetal bovine serum (FBS; Ausgenex, Molendinar, Australia), and 1% antibiotic–antimycotic solution (AA; Gibco). The cell suspension was passed through a 100‐μm cell strainer (Becton Dickinson and Company [BD], Franklin Lakes, NJ) and washed in 1 × Dulbecco's phosphate‐buffered saline (PBS; Gibco). Primary chondrocytes were suspended in Cellbanker 1 cryopreservation medium (Zenoaq, Fukushima, Japan) and cryopreserved at −80°C. Synoviocytes were cultured to Passage 1 and preserved in the same manner as the chondrocytes. The frozen chondrocytes and synoviocytes were thawed, and the chondrocytes were seeded on temperature‐responsive culture inserts (UpCell® 4.2 cm^2^, CellSeed, Tokyo, Japan) at 50,000 cells/cm^2^, whereas the synoviocytes were seeded on six‐well companion plates (Corning, Corning, NY, USA) at 10,000 cells/cm^2^, followed by coculturing. The culture medium was initially composed of DMEM/F12, 20% FBS, and 1% AA. The medium was changed every 3 or 4 days thereafter, with the addition of 100 μg/ml ascorbic acid (Wako Pure Chemical Industries, Osaka, Japan). After coculturing for 2 weeks, three chondrocyte sheets were layered using a polyvinylidene difluoride membrane and were cultured for another 7 days. The synoviocytes were also collected in sheet forms and were cultured for another 7 days. To count the cells, fabricated LC sheets and SY sheets were digested enzymatically, and trypan blue exclusion assays were performed. LC sheets and SY sheets were cut appropriately just prior to transplantation so that they contained approximately 5.0 × 10^5^ cells.

### 2.2 Histological and immunohistochemical analysis of LC sheets and SY sheets

For histological analysis, LC sheets and SY sheets were fixed in 4% paraformaldehyde in 0.01 M PBS and embedded in optimum cutting temperature compound (Sakura Finetek, Tokyo, Japan). Frozen 10 μm sections were stained with 0.1% Safranin O aqueous solution (Chroma Gesellschaft Schmid & Co., Munster, Germany) and 0.08% fast green aqueous solution (Chroma Gesellschaft Schmid & Co., Munster, Germany). The sections were also stained with hematoxylin (Merck Millipore, Darmstadt, Germany) and eosin (Muto pure chemicals Co., Ltd., Tokyo, Japan; HE). For immunohistochemical analysis, 20 μm frozen sections were blocked with 5% normal goat serum (NGS; Rockland Immunochemicals, Limerick, PA, USA) and 0.3% TritonX‐100 (Sigma‐Aldrich, St. Louis, MO, USA) for 30 min, then incubated at 4°C overnight with primary antibodies to type I collagen (COL I; Southern Biotech, Birmingham, AL, USA; dilution, 1:200); type II collagen (COL II; Kyowa Pharma Chemical, Toyama, Japan; dilution 1:200), or fibronectin (Merck Millipore, Darmstadt, Germany; dilution 1:500). The sections were washed and incubated at room temperature (RT) for 1 h with secondary antibody goat anti‐mouse immunoglobulin (Ig) Alexa Fluor 488 (Thermo Fisher Scientific, Waltham, MA, USA) for COL II and fibronectin, and donkey anti‐goat Ig Alexa Fluor 546 (Thermo Fisher Scientific) for COL I. The slides were then stained with 4′,6‐diamidino‐2‐phenylindole (DAPI; Vector Laboratories, Burlingame, CA, USA), and observed under a fluorescence microscope (BZ‐9000 Generation II, Keyence Corp., Osaka, Japan).

### 2.3 RNA extraction and reverse transcription–PCR

LC sheets and SY sheets were incubated with TRIzol (Thermo Fisher Scientific) for RNA extraction. Total RNA was extracted using the RNeasy Mini Kit (Qiagen Inc., Valencia, CA, USA), according to the manufacturer's instructions. cDNA was synthesized using the QuantiTect Reverse Transcription Kit (Qiagen Inc., Hilden, Germany) at 42°C for 15 min and 95°C for 3 min. Table 1 lists the oligonucleotide primers used. Polymerase chain reaction (PCR) was performed using SYBR Green PCR Master Mix (Applied Biosystems, Foster City, CA, USA) and the cDNA template (2.5 ng) in a total reaction volume of 25 μl using a 7300 Real‐time PCR system (Applied Biosystems) at 95°C for 15 s, 60°C for 60 s, for 35–45 cycles. The results were analyzed using Smart Cycler II software (Applied Biosystems). The internal control glyceraldehyde‐3‐phosphate dehydrogenase was used as a reference gene, and the comparative 2^–ΔΔCt^ method was used for analysis.

**TABLE 1:** Primers used for reverse‐transcriptase–polymerase chain reaction

Target genes	Primer sequence: forward	Primer sequence: reverse
GAPDH	5′‐ACCCAGAAGACTGTGGATGG‐3′	5′‐TTCTAGACGGCAGGTCAGGT‐3′
COL1A1	5′‐GTCGAGGGCCAAGACGAAG‐3′	5′‐CAGATCACGTCATCGCACAAC‐3′
COL1A2	5′‐AATTGGAGCTGTTGGTAACGC‐3′	5′‐CACCAGTAAGGCCGTTTGC‐3′
COL2A1	5′‐GTGGAGCAGCAAGAGCAA‐3′	5′‐TGTTGGGAGCCAGATTGT‐3′
COL10A1	5′‐ATGCTGCCACAAATACCCTTT‐3′	5′‐GGTAGTGGGCCTTTTATGCCT‐3′
SOX9	5′‐AACGCCGAGCTCAGCAAGA‐3′	5′‐CCGCGGCTGGTACTTGTAATC‐3′
MMP13	5′‐ACTGAGAGGCTCCGAGAAATG‐3′	5′‐GAACCCCGCATCTTGGCTT‐3′
ACAN	5′‐AGGAGACAGAGGGACACGTC‐3′	5′‐TCCACTGGTAGTCTTGGGCAT‐3′
RUNX2	5′‐ACCATGGTGGAGATCATCG‐3′	5′‐CGCCATGACAGTAACCACAG‐3′

*Note*: Primer sequences for COL1A1, COL1A2, and COL10A1 from Yamashita et al. (2015).

Abbreviations: ACAN, aggrecan; COL10A1, collagen, type X, alpha 1; COL1A1, collagen, type I, alpha 1; COL1A2, collagen, type I, alpha 2; COL2A1, collagen, type II, alpha 1; GAPDH, glyceraldehyde‐3‐phosphate dehydrogenase; MMP13, matrix metalloproteinase 13; RUNX2, Runt‐related transcription factor 2; SOX9, SRY‐Box 9.

### 2.4 Measurement of humoral factors

A random selection of LC sheets and SY sheets were further cultured for 72 h in 3 ml of DMEM/F12, supplemented with 1% FBS and 1% AA incubated at 37°C under appropriate cell culture condition. Supernatants were collected and centrifuged at 15,000 *g* for 10 min to remove cell debris. The concentrations of transforming growth factor‐β‐1 (TGF‐β1; R&D Systems), and melanoma inhibitory activity (MIA; Roche, Basel, Switzerland) were measured using enzyme‐linked immunosorbent assay (ELISA) kits. The signal detected for blank medium containing 1% FBS was subtracted to adjust for the protein content of the FBS. Measurements were repeated at least twice for each donor, and averages are reported. The concentrations of TGF‐β1 or MIA were normalized by the number of cells (1.0 × 10^6^ cells).

### 2.5 Transplantation of LC sheets and SY sheets

Forty‐eight 12‐week‐old rats (F334/NJcl‐rnu/rnu; Clea Japan, Tokyo, Japan), weighing approximately 270 g, were used in the transplantation experiments. The animals were housed two animals per cage and were given daily standard chow and access to water ad libitum. LC sheets and SY sheets fabricated from each of the donors were allocated equally to each transplantation group. The rats were anesthetized using isoflurane, N_2_O, and O_2_. An osteochondral defect was created using the methods described by Itokazu et al. (2016). Briefly, a medial parapatellar incision was made on the right knee; the patella was moved laterally and an osteochondral defect (diameter 2 mm; depth 1 mm) was created on the patellar groove of the femur using a biopsy punch (Kai Industries, Seki, Japan) and hand drill. Marrow bleeding during creation of the osteochondral defect was confirmed. Next, LC sheets and SY sheets were transplanted as: Group A, untreated (without transplantation); Group B, LC sheet containing 5.0 × 10^5^ cells; Group C, SY sheet containing 5.0 × 10^5^ cells, and Group D: both SY and LC sheets, each containing 5.0 × 10^5^ cells. For Group D, the SY sheet was transplanted first and then covered with the LC sheet. The patella was anatomically repositioned and the quadriceps femoris muscle, tendon, and skin were sutured. After the surgery, all the rats were returned to their cages without splinting or immobilization.

### 2.6 Pain evaluation

An incapacitance tester (Linton Instrumentation, Norfolk, UK) was used to evaluate the degree of pain, inflammation, or discomfort as previously reported (Ito et al., 2012; Mihara, Higo, Uchiyama, Tanabe, & Saito, 2007; Takahashi et al., 2018; Tani et al., 2017). All animals were acclimated to the incapacitance tester for 7 days prior to transplantation. Nine measurements were taken, on days 1, 5, 8, 12, 15, 19, 22, 26, and 28 after transplantation. The average damaged‐limb weight distribution ratio (%) of the hind limbs was calculated from 10 repeated measurements for each animal and averaged for each group as: Damaged‐limb weight distribution ratio (%) = damaged‐limb load (g)/total limb load (g) × 100.

### 2.7 Histological evaluation of repaired cartilage

The animals were euthanized by administration of high‐dose anesthesia at 4 or 12 weeks after transplantation. The operated knee was opened, and the distal portion of the femur was excised and fixed for 1 week in 20% formalin (Wako Pure Chemical Industries). The sample was decalcified in 10% EDTA (Wako Pure Chemical Industries) for 2 and 3 weeks and embedded in paraffin wax, and 8 μm sections were cut near the center of the defect area, parallel to the long axis of the femur. Standard protocols were used for histological staining. Deparaffinized sections were stained with 0.1% Safranin O aqueous solution (Chroma Gesellschaft Schmidt & Co., Munster, Germany) and 0.08% fast green aqueous solution (Chroma Gesellschaft Schmid & Co., Munster, Germany). These were randomized and scored separately by two trained orthopedic surgeons (A.T. and Y.S.), who were blinded to their identities, using the International Cartilage Repair Society (ICRS) histological grading system (Brehm et al., 2006; Mainil‐Varlet et al., 2003; O'Driscoll, Keeley, & Salter, 1988), which is a modification of that reported by O'Driscoll et al. (1988). This system evaluates repaired tissue based on 11 items: tissue morphology, matrix staining, structural integrity, cluster formation, tidemark opening, bone formation, histological appraisal of surface architecture, histological appraisal of the degree of defect filling, lateral integration of defect‐filling tissue, basal integration of defect‐filling tissue, and histological signs of inflammation. Eleven items were evaluated by scoring as shown in Table 2. The total score ranged from 11 points (no repair) to 45 points (normal articular cartilage).

**TABLE 2:** ICRS histological grading system

Item	Description
Ti	1: Exclusively noncartilage
	2: Mostly noncartilage
	3: Mostly fibrocartilage
	4: Mostly hyaline cartilage
Matx	1: None
	2: Slight
	3: Moderate
	4: Strong
Stru	1: Severe disintegration
	2: Cysts or disruptions
	3: No organization of chondrocytes
	4: Beginning of columnar organization of chondrocytes
	5: Normal, similar to healthy mature cartilage
Clus	1: 25–100% of the cells clustered
	2: <25% of the cells clustered
	3: No clusters
Tide	1: <25% of the calcified cartilage layer intact
	2: 25–49% of the calcified cartilage layer intact
	3: 50–75% of the calcified cartilage layer intact
	4: 76–90% of the calcified cartilage layer intact
	5: Complete intactness of the calcified cartilage layer
Bform	1: No formation
	2: Slight
	3: Strong
SurfH	1: Severe fibrillation or disruption
	2: Moderate fibrillation or irregularity
	3: Slight fibrillation or irregularity
	4: Normal
FilH	1: <25%
	2: 26–50%
	3: 51–75%
	4: 76–90%
	5: 91–110%
Latl	1: Not bonded
	2: Bonded at one end/partially both ends
	3: Bonded at both sides
Basl	1: <50%
	2: 50–70%
	3: 70–90%
	4: 91–100%
InfH	1: Strong inflammation
	3: Slight inflammation
	5: No inflammation
Hgtot	45 points

*Note*: This system evaluates repaired tissue based on 11 items: Ti: tissue morphology; Matx: matrix staining; Stru: structural integrity; Clus: cluster formation; Tide: tidemark opening; Bform: bone formation; SurfH: histological appraisal of surface architecture; FilH: histological appraisal of the degree of defect filling; Latl: lateral integration of defect‐filling tissue; Basl: basal integration of defect‐filling tissue; InfH: histological signs of inflammation. The total score (Hgtot) range is 11–45. ICRS: International Cartilage Regeneration and Joint Preservation Society.

To immunostain for COL I, deparaffinized sections were treated with 0.01 M citric acid buffer for 10 min at 98°C; to immunostain for COL II, deparaffinized sections were treated with 0.4% pepsin for 30 min at 37°C. The sections were washed in distilled water, blocked with normal equine serum for 10 min at RT, and then treated with primary antibodies to COL I (Southern Biotech; dilution 1:100) or COL II (Kyowa Pharma Chemical; dilution 1:100) for 3 h at RT. The stained sections were washed in 0.01 M PBS, treated with ImmPRESS polymer anti‐mouse IgG reagent (Vector Laboratories) for 1 h at RT, immersed for 2–4 min in Tris–HCl buffer (pH 7.6) containing 0.05% diaminobenzidine and 0.005% hydrogen peroxide, and then counterstained with HE.

To immunostain for human vimentin (hVIM), deparaffinized sections were treated with target retrieval solution (S1699; Dako, Glostrup, Denmark) for 30 min at 98°C and then cooled for 30 min. The sections were washed in distilled water, blocked with 5% NGS for 10 min at RT, and then treated with primary antibody to hVIM (ab16700; Abcam, Cambridge, MA, USA; dilution 1:100) overnight at 4°C. The stained sections were washed in 0.01 M PBS, treated with secondary antibody gout anti‐rabbit Ig Alexa Fluor 488 (Thermo Fisher Scientific) for 1 h at RT, and then stained with DAPI. All microscopic images were obtained using a fluorescence microscope (BZ‐9000 Generation II, Keyence Corp., Osaka, Japan).

### 2.8 Statistical analysis

The results are presented as mean ± standard deviation, and *p* < 0.05 was deemed significant. Statistical analysis was performed using SPSS v23.0 software (IBM Corp., Armonk, NY, USA). The reverse transcription PCR and ELISA results were analyzed using the Mann–Whitney *U* test. Analysis of variance was used to analyze the weight distribution ratios and ICRS scores, and the Tukey honest significant difference (HSD) method was used for post hoc tests.

## 3 RESULTS

### 3.1 Properties of LC sheets and SY sheets

**FIGURE 1:**
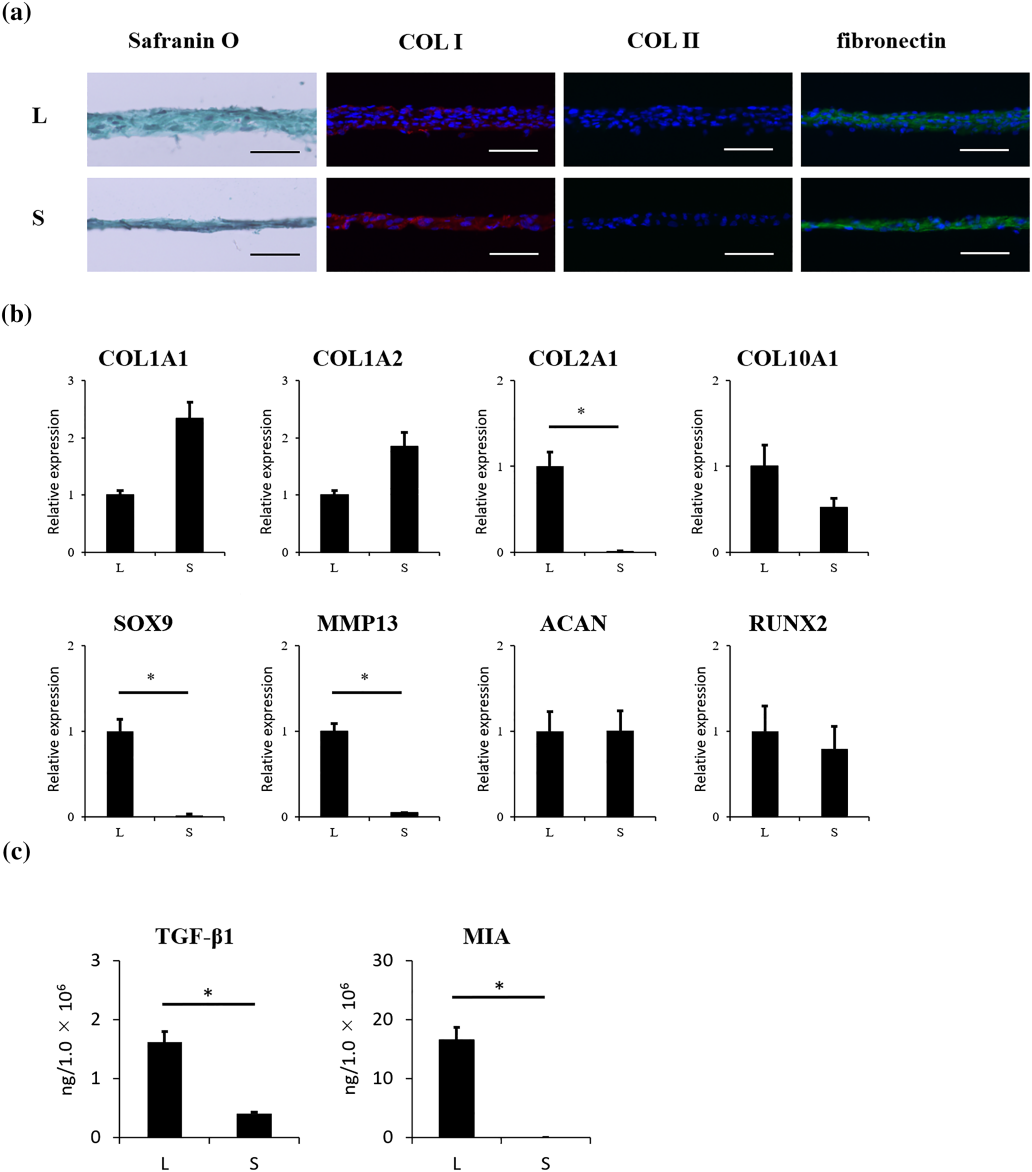
Properties of LC sheets and SY sheets. For each sheet, *n* = 6. (a) Histological analysis of LC sheets and SY sheets revealed negative staining for Safranin O. Immunohistochemical analysis revealed positive staining for COL I and fibronectin, but negative staining for COL II for both sheet types. Scale bar = 50 μm. (b) Gene expression profile of LC sheets and SY sheets. mRNA expression for COL2A1, SOX9, and MMP13 was significantly higher in LC sheets than in SY sheets (*p* < 0.05). (c) The concentrations of humoral factors secreted by LC sheets and SY sheets. The secretion of TGF‐β1 was 1.6 ± 0.2 ng/1.0 × 10^6^ cells by LC sheets and 0.40 ± 0.02 ng/1.0 × 10^6^ cells by SY sheets. The secretion of MIA by LC sheets was 16.5 ± 2.1 ng/1.0 × 10^6^ cells, compared with undetectable levels secreted by SY sheets. The secretion of MIA and TGF‐β1 by LC sheets was significantly higher than that by SY sheets (*p* < 0.05). The results are presented as mean ± standard deviation. LC: layered chondrocyte; SY: synoviocyte; COL I: type I collagen; COL II: type II collagen; COL1A1: collagen, type I, alpha 1; COL1A2: collagen, type I, alpha 2; COL2A1: collagen, type II, alpha 1; COL10A1: collagen, type X, alpha 1; SOX9: SRY‐Box 9; MMP13: matrix metalloproteinase 13; ACAN: aggrecan; RUNX2: Runt‐related transcription factor 2; TGF‐β1: transforming growth factor‐β‐1; MIA: melanoma inhibitory activity; L: LC sheets; S: SY sheets [Colour figure can be viewed at wileyonlinelibrary.com]

An average LC sheet and SY sheet contained 1.3 ± 0.3 × 10^6^ cells and 1.4 ± 0.2 × 10^6^ cells, respectively. LC sheets and SY sheets stained negative for Safranin O and COL II and positive for COL I and fibronectin; no differences in staining patterns were detected between the two sheet types (Figure 1a). The expression of mRNA for collagen, type II, alpha 1 (COL2A1), SRY‐Box 9 (SOX9), and matrix metalloproteinase 13 (MMP13) was significantly higher in LC sheets than in SY sheets (Figure 1b). The concentrations of humoral factors secreted by LC sheets and SY sheets are summarized in Figure 1c. Compared with SY sheets, LC sheets secreted higher concentrations of TGF‐β1 (LC sheets 1.6 ± 0.2 ng/1.0 × 10^6^ cells; SY sheets 0.40 ± 0.02 ng/1.0 × 10^6^ cells) and MIA (LC sheets 16.5 ± 2.1 ng/1.0 × 10^6^ cells; SY sheets were below the detection limit).

### 3.2 Pain evaluation

**FIGURE 2:**
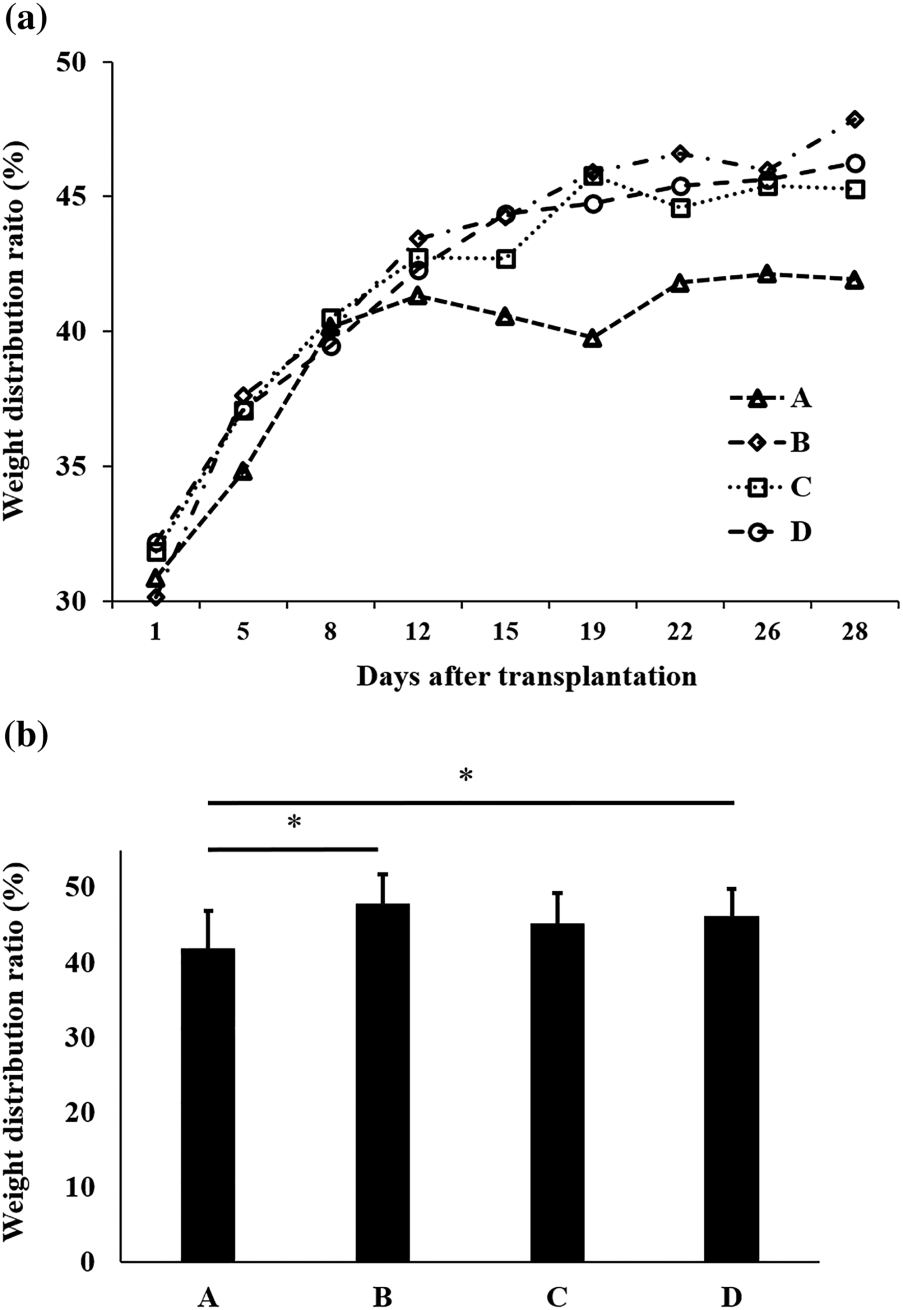
Pain evaluation. (a) Weight distribution ratios of the damaged limb after transplantation. Group A, untreated; Group B, LC sheet containing 5.0 × 10^5^ cells alone; Group C, SY sheet containing 5.0 × 10^5^ cells alone; and Group D, SY sheet plus LC sheet, each containing 5.0 × 10^5^ cells. For each group, *n* = 6. Group A showed minimal improvement. Groups B, C, and D all showed improvement. (b) Weight distribution ratios on Day 28 after transplantation: Group A = 41.9 ± 5.0%, Group B = 47.9 ± 3.9%, Group C = 45.3 ± 4.0%, and Group D = 46.3 ± 3.6%. The weight distribution ratios of Groups B and D were significantly higher than that of Group A (*p* < 0.05). The results are presented as mean ± standard deviation. LC: layered chondrocyte; SY: synoviocyte

The weight distribution ratio was used as a measure of pain and was tested daily for 28 days after transplantation (Figure 2a). Group A showed minimal improvement from Day 1 to Day 28: 30.9 ± 6.8% to 41.9 ± 5.0%. Groups B, C, and D all showed improvement from Day 1 to Day 28: 30.1 ± 5.3% to 47.9 ± 3.9% in Group B, 31.8 ± 4.5% to 45.3 ± 4.0% in Group C, and 32.2 ± 6.4% to 46.3 ± 3.6% in Group D. The weight distribution ratios for Day 28 were significantly higher for Groups B and D than for Group A, as shown in Figure 2b (*p* < 0.05).

### 3.3 Microscopic analysis of repaired tissue

**FIGURE 3:**
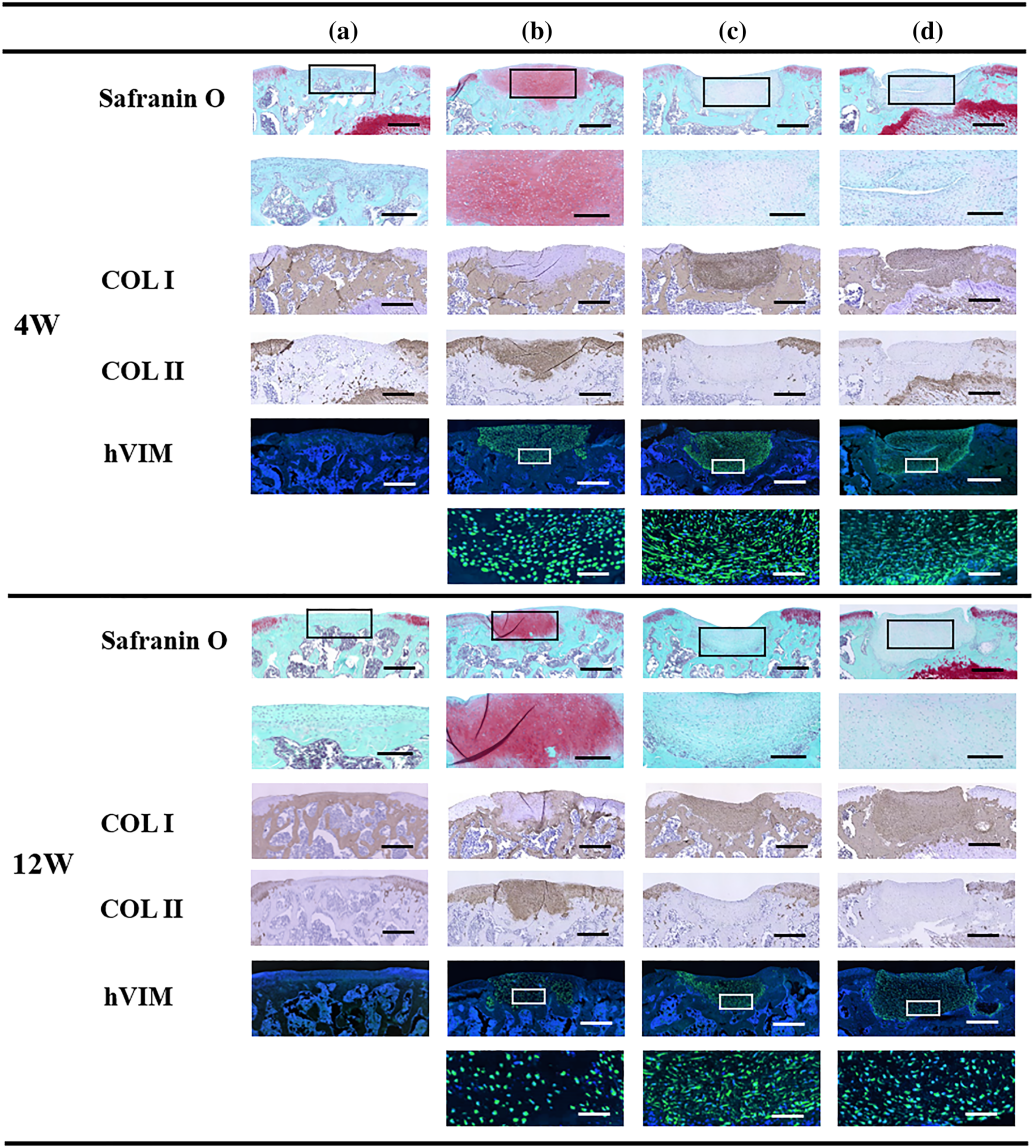
Representative microscopic images from the histological and immunohistochemical analyses. Group A, untreated; Group B, LC sheet containing 5.0 × 10^5^ cells alone; Group C, SY sheet containing 5.0 × 10^5^ cells alone; and Group D, SY sheet plus LC sheet, each containing 5.0 × 10^5^ cells. For each group, *n* = 6. Four weeks after transplantation in Groups B, C, and D, the defects were filled with repaired tissue. Histological analysis of Group A revealed no Safranin O staining or formation of a chondral layer but revealed bone‐like tissue or fibrous tissue. In Group B, strong Safranin O staining was observed. In Groups C and D, no Safranin O staining was observed. Immunohistochemical analysis revealed negative staining for COL II and hVIM in Group A, positive staining for COL II and hVIM in Group B, and positive staining for COL I and hVIM in Groups C and D. At 12 weeks after transplantation, the defects in Groups B, C, and D, in which transplantation was performed, were filled with repaired tissue. Histological analysis of Group A revealed no Safranin O staining or formation of a chondral layer but revealed bone‐like tissue. In Group B, strong Safranin O staining was observed. In Groups C and D, no Safranin O staining was observed. Immunohistochemical analysis revealed negative staining for COL II and hVIM in Group A, positive staining for COL II and hVIM and partial staining for COL I in Group B, and positive staining for COL I and hVIM in Groups C and D. Low‐power images of Safranin O, COL I, COL II, and hVIM staining are shown in the upper rows (scale bar = 500 μm). High‐power images of Safranin O and hVIM are shown in the lower rows (scale bar of Safranin O = 200 μm, scale bar of hVIM = 50 μm). LC: layered chondrocyte; SY: synoviocyte; COL I: type I collagen; COL II: type II collagen; hVIM: human vimentin [Colour figure can be viewed at wileyonlinelibrary.com]

**FIGURE 4:**
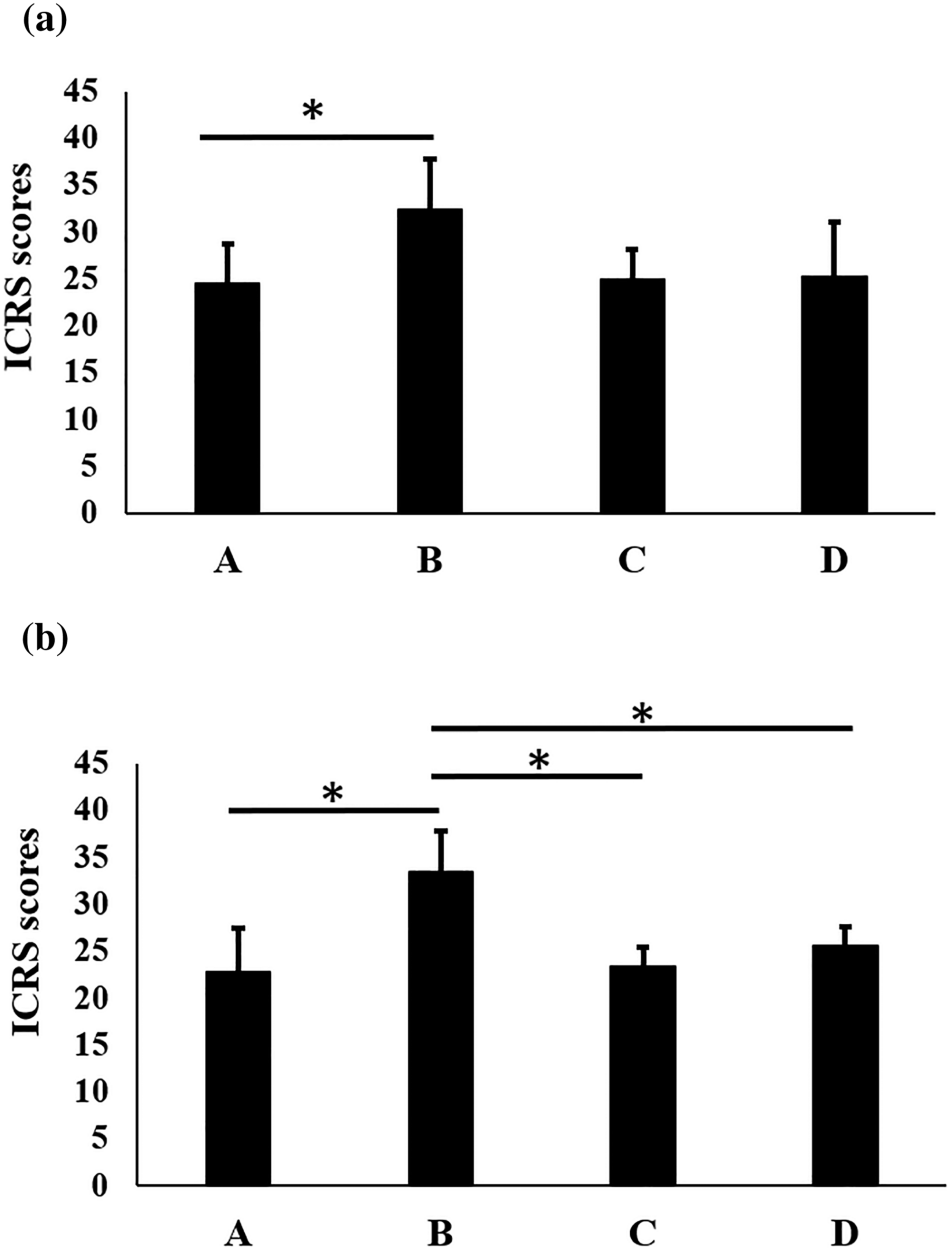
Histological evaluation of repaired tissue at (a) 4 weeks and (b) 12 weeks after transplantation. Group A, untreated; Group B, LC sheet containing 5.0 × 10^5^ cells alone; Group C, SY sheet containing 5.0 × 10^5^ cells alone; and Group D, SY sheet plus LC sheet, each containing 5.0 × 10^5^ cells. For each group, *n* = 6. At 4 weeks after transplantation, the scores for Groups A, B, C, and D were 24.6 ± 4.1, 32.3 ± 5.4, 24.9 ± 3.2, and 25.2 ± 5.9, respectively. ICRS scores in Group B were significantly higher than those in Group A (*p* < 0.05). At 12 weeks after transplantation, the scores for Groups A, B, C, and D were 22.8 ± 4.6, 33.5 ± 4.4, 23.3 ± 2.1, and 25.5 ± 2.1, respectively. ICRS scores in Group B were significantly higher than those in Groups A, C, and D (*p* < 0.05). The results are presented as mean ± standard deviation. LC: layered chondrocyte; SY: synoviocyte; ICRS: International Cartilage Regeneration and Joint Preservation Society

No signs of infection or rejection were observed in any group during the experiment. Safranin O staining was performed to evaluate the repaired tissue, as shown in Figure 3. Four weeks after transplantation, the defects in Groups B, C, and D were filled with repaired tissue. In Group A, the defects were not filled with a chondral layer, but with either bone‐like tissue or fibrous tissue that did not stain for Safranin O or COL II staining but did stain for COL I. In Group B, the repaired tissue showed Safranin O and COL II staining and no COL I staining, good integration with the surrounding cartilage, a high defect‐filling rate, and a smooth superficial layer, although the subchondral bone formation was inadequate. In Groups C and D, the repaired tissue showed good integration with the surrounding cartilage, but the repaired tissue was mostly fibrous tissue, with no Safranin O or COL II staining and with COL I staining, and the superficial layer and subchondral bone formation were inadequate. At 12 weeks after transplantation, the defects in Groups B, C, and D were filled with repaired tissue. In Group A, the defects were filled with bone‐like tissue. In Group B, the repaired tissue showed Safranin O and COL II staining and partial COL I staining, integration with the surrounding tissue, a high defect‐filling rate, a smooth superficial layer, and a columnar arrangement of the chondrocytes. There was moderate formation of subchondral bone. In Groups C and D, the repaired tissue had not changed significantly from that which was present 4 weeks after transplantation. A modified version of the ICRS grading system was used to evaluate cartilage repair (Table 2). Figure 4 and Table 3 show the ICRS grading system results 4 and 12 weeks after transplantation. At 4 weeks after transplantation, the scores for Groups A, B, C, and D were 24.6 ± 4.1, 32.3 ± 5.4, 24.9 ± 3.2, and 25.2 ± 5.9, respectively. The score for Group B was significantly higher than that for Group A. At 12 weeks after transplantation, the scores for Groups A, B, C, and D were 22.8 ± 4.6, 33.5 ± 4.4, 23.3 ± 2.1, and 25.5 ± 2.1, respectively. The score for Group B was significantly higher than those for Groups A, C, and D.

**TABLE 3:** ICRS grades 4 and 12 weeks after transplantation

	Item	A	B	C	D
4W	Ti	2.1 ± 0.7	3.5 ± 0.5	1.8 ± 0.2	2.1 ± 0.6
	Matx	1.3 ± 0.4	2.4 ± 0.8	1.8 ± 0.3	1.6 ± 0.5
	Stru	2.3 ± 0.6	3.7 ± 0.5	1.9 ± 0.1	2.4 ± 1.1
	Clus	1.0 ± 0.0	1.1 ± 0.1	1.0 ± 0.0	1.0 ± 0.0
	Tide	1.0 ± 0.0	2.2 ± 1.1	1.0 ± 0.0	1.2 ± 0.4
	Bform	2.4 ± 0.4	2.3 ± 0.4	1.0 ± 0.0	1.4 ± 0.6
	SurfH	2.4 ± 0.6	2.8 ± 0.8	2.0 ± 0.3	2.0 ± 0.6
	FilH	3.5 ± 0.9	3.9 ± 0.9	3.6 ± 0.9	3.3 ± 1.3
	Latl	1.7 ± 0.8	2.4 ± 0.5	2.4 ± 0.6	1.9 ± 0.7
	Basl	1.9 ± 0.8	3.1 ± 1.0	3.4 ± 1.1	2.7 ± 0.9
	InfH	5.0 ± 0.0	5.0 ± 0.0	5.0 ± 0.0	5.0 ± 0.0
	Hgtot	24.6 ± 4.1	32.3 ± 5.4	24.9 ± 3.2	25.2 ± 5.9
	Item	A	B	C	D
12W	Ti	1.7 ± 0.4	3.0 ± 0.9	1.9 ± 0.1	1.9 ± 0.1
	Matx	1.2 ± 0.4	2.4 ± 1.3	1.3 ± 0.5	1.5 ± 0.5
	Stru	2.2 ± 0.7	2.9 ± 0.8	1.9 ± 0.5	2.2 ± 0.3
	Clus	1.1 ± 0.1	1.1 ± 0.1	1.0 ± 0.0	1.0 ± 0.0
	Tide	1.0 ± 0.0	2.2 ± 1.2	1.0 ± 0.0	1.0 ± 0.0
	Bform	2.8 ± 0.2	2.6 ± 0.5	1.3 ± 0.6	1.4 ± 0.5
	SurfH	2.1 ± 0.6	3.1 ± 0.5	1.9 ± 0.5	2.4 ± 0.6
	FilH	3.4 ± 0.8	5.0 ± 0.0	3.1 ± 0.6	3.6 ± 0.7
	Latl	1.5 ± 0.8	2.6 ± 0.4	1.7 ± 0.5	1.9 ± 0.6
	Basl	1.5 ± 0.6	3.6 ± 0.6	3.1 ± 0.6	3.3 ± 0.6
	InfH	5.0 ± 0.0	5.0 ± 0.0	5.0 ± 0.0	5.0 ± 0.0
	Hgtot	22.8 ± 4.6	33.5 ± 4.4	23.3 ± 2.1	25.5 ± 2.1

*Note*: Group A, untreated; Group B, LC sheet containing 5.0 × 10^5^ cells alone; Group C, SY sheet containing 5.0 × 10^5^ cells alone; and Group D, SY sheet plus LC sheet, each containing 5.0 × 10^5^ cells. LC: layered chondrocyte; SY: synoviocyte. For each group, *n* = 6. ICRS: International Cartilage Regeneration and Joint Preservation Society; Ti: tissue morphology; Matx: matrix staining; Stru: structural integrity; Clus: cluster formation; Tide: tidemark opening; Bform: bone formation; SurfH: histological appraisal of surface architecture; FilH: histological appraisal of the degree of defect filling; Latl: lateral integration of defect‐filling tissue; Basl: basal integration of defect‐filling tissue; InfH: histological signs of inflammation. The total score (Hgtot) range is 11–45.

### 3.4 Localization of human‐derived cells in repaired tissue

Figure 3 shows the immunostaining for hVIM. Groups B, C, and D showed positive hVIM staining at 4 weeks after transplantation, but the overall number of positive cells decreased at 12 weeks after transplantation.

## 4 DISCUSSION

Appropriate translational models that can directly evaluate the final outcomes are necessary to confirm the safety and efficacy of human cell‐derived products. In the present study, LC sheets, similar to those used in our previous clinical study, and SY sheets fabricated from synovium‐derived cells, were evaluated in vitro and in vivo.

LC sheets and SY sheets were both negative for Safranin O staining and positive for fibronectin and COL I immunostaining. Compared with the chondrocyte sheets fabricated from the surgical waste material of younger patients (Kokubo et al., 2016), the LC sheets used in this study were fabricated from older patients, which may explain why these LC sheets did not stain for Safranin O or COL II. Gene expression analysis revealed that compared with SY sheets, LC sheets had significantly higher expression of COL2A1 and SOX9 suggesting that they had better chondrogenic properties. MMP13 expression was significantly higher in LC sheets than in SY sheets. In previous study, the LC sheets restrain the catabolic factors matrix metalloproteinase 3, MMP13, and ADAMTS5 in allogenic transplantation on rabbit model (Kaneshiro et al., 2006). Also, MMP13 expression did not differ significantly between LC sheets and layered SY sheets, and that ADAMTS4 and 5 were significantly higher in the layered SY sheets than the LC sheets in allogenic transplantation on rat model (Shimizu et al., 2015). The difference is that the cells for the current study were collected from older patients with knee OA. Furthermore, collagen, type X, alpha 1 expression in this study was higher in the LC sheets than in SY sheets. Thus, it is possible that LC sheets may be particularly affected by OA.

ELISA analysis revealed that LC sheets produced significantly higher levels of TGF‐β1 and MIA, which have, respectively, been suggested to make important contributions to cartilage repair and to be a marker for the cartilage phenotype.

Cartilage repair was observed at 4 and 12 weeks after transplantation of LC sheets, with effects including alleviation of pain and an improvement in histological scores. To our knowledge, this is the first study in which human chondrocyte sheets were transplanted to immune‐deficient rats and cartilage repair was observed. Consistent with the reported by Takahashi et al. (2018), the repaired tissue in Group B contained a mixture of hyaline cartilage and fibrocartilage, possibly because the cells were collected from older patients with knee OA. We previously reported the high gene expression of fibronectin, integrin α10, other cell adhesion factors, SOX9 (a key regulator of chondrocyte differentiation), and COL2A1 and collagen, type XXVII, alpha 1 in LC sheets, indicating that these sheets have excellent adhesiveness and maintenance of the cartilage phenotype (Mitani et al., 2009). In the previous study, we confirmed that LC sheets secrete MIA, TGF‐β1, and prostaglandin E2, which are anabolic factors involved in cartilage repair (Hamahashi et al., 2015).

Cartilage repair was not observed in Groups C and D at 4 or 12 weeks after transplantation, and the osteochondral defects were filled with fibrous tissue. Ito et al. (2012) reported that, in rabbits, better cartilage regeneration was achieved by transplanting a combination of LC sheets and SY sheets than by LC sheets or SY sheets alone. Shimizu et al. (2015) reported that in rats, the transplantation of SY sheets as a monolayer or a triple layer did not promote hyaline cartilage repair. Our results show that SY sheets, alone or in combination with LC sheets at a ratio of 1:1, did not promote hyaline cartilage repair. These differences may relate to the differing characteristics of SY sheets from different animal species (rabbits, rats, or humans) and the fact that, in our study, the cells were collected from older patients with knee OA.

At 4 and 12 weeks after transplantation, immunostaining with hVIM showed that transplanted cells were still present in the osteochondral defects. However, at 12 weeks after transplantation, the number of transplanted cells had decreased. This finding is consistent with those of Itokazu et al. (2016), who reported that the number of transplanted cells had decreased 12 weeks after transplantation of xenogeneic cells to immune‐deficient rats, and with those of Takaku et al. (2014), who reported that after transplantation of allogeneic chondrocyte sheets in rats, cells derived from the transplants mostly disappeared within the joints at 4–8 weeks after transplantation, although cells were still detected 21 months after transplantation. Although cartilage is a tissue with relatively high immunological tolerance, it is likely that immunosuppression is required for xenogeneic transplantation. Takahashi et al. (2018) performed xenogeneic transplantation of human chondrocyte sheets into an immune‐deficient rabbit under immunosuppression. Although they confirmed that transplanted cells were present at 4 weeks after transplantation, they reported immune rejection at 12 weeks after transplantation, when the effects of the immunosuppression wore off. In the present study, we used F334/NJcl‐rnu/rnu rats for xenogeneic transplantation experiments. These rats have an immunological deficit in T‐cell function and are not likely to reject transplanted tissues; therefore, replacement of transplanted cells by host‐derived cells secondary to chondrogenesis and time is believed to be the reason for the decreased number of cells detected.

There were two limitations to this study. First, 12 weeks posttransplantation is a short period for evaluating cartilage repair. Therefore, evaluation over a longer term is warranted. Second, this study was conducted on small animals. The rat model is low cost and suitable for the selection of cell sources; however, validation of the efficacy of transplantation is required in larger animals that more closely resemble humans.

Evaluation of human chondrocyte sheets using immune‐deficient rats was possible for up to 12 weeks after transplantation. The in vitro evaluation revealed that human chondrocyte sheets with a cartilage‐specific phenotype contributed to cartilage regeneration in vivo. We plan to verify the efficacy of various cell sources for chondrocyte sheets using this translational model.

## CONFLICT OF INTEREST

M.S. receives research funds from CellSeed Inc.

## AUTHOR CONTRIBUTIONS

D.T.: Lead for all in vivo and in vitro elements of experimental work and manuscript preparation. M.S.: Guarantor, contributions to experimental design, lead for all elements of experimental work, provision of funding, and manuscript proofing. E.O.: Contributions to experimental design and in vivo elements of experimental work. T.T.: Contributions to experimental design, in vivo elements of experimental work, statistical analysis, and manuscript proofing. M.M.: Contributions to in vitro elements of experimental work. A.T.: Contributions to in vivo elements of experimental work. Y.S.: Contributions to in vivo elements of experimental work. E.T.: Contributions to experimental design and in vitro elements of experimental work. M.W.: Contributions to experimental design and manuscript proofing.

## References

[term3101-bib-0001] Brehm, W. , Aklin, B. , Yamashita, T. , Rieser, F. , Trüb, T. , Jakob, R. P. , & Mainil‐Varlet, P. (2006). Repair of superficial osteochondral defects with an autologous scaffold‐free cartilage construct in a caprine model: Implantation method and short‐term results. Osteoarthritis and Cartilage, 14, 1214–1226. 10.1016/j.joca.2006.05.002 16820305

[term3101-bib-0002] Brittberg, M. , Lindahl, A. , Nilsson, A. , Ohlsson, C. , Isaksson, O. , & Peterson, L. (1994). Treatment of deep cartilage defects in the knee with autologous chondrocyte transplantation. New England Journal of Medicine, 331, 889–895. 10.1056/NEJM199410063311401 8078550

[term3101-bib-0003] Brittberg, M. , Gomoll, A. H. , Canseco, J. A. , Far, J. , Lind, M. , & Hui, J. (2016). Cartilage repair in the degenerative ageing knee. Acta Orthopaedica, 87(sup363), 26–38. 10.1080/17453674.2016.1265877 27910738 PMC5389429

[term3101-bib-0004] Buckwalter, J. A. , & Mankin, H. J. (1998). Articular cartilage: Degeneration and osteoarthritis, repair, regeneration, and transplantation. Instructional Course Lectures, 47, 487–504. 9571450

[term3101-bib-0005] Buckwalter, J. A. , Mankin, H. J. , & Grodzinsky, A. J. (2005). Articular cartilage and osteoarthritis. Instructional Course Lectures, 54, 465–480. 15952258

[term3101-bib-0006] Bugbee, W. D. , Pallante‐Kichura, A. L. , Görtz, S. , Amiel, D. , & Sah, R. (2016). Osteochondral allograft transplantation in cartilage repair: Graft storage paradigm, translational models, and clinical applications. Journal of Orthopaedic Research : Official Publication of the Orthopaedic Research Society, 34(1), 31–38. 10.1002/jor.22998 26234194 PMC4732516

[term3101-bib-0007] Ebihara, G. , Sato, M. , Yamato, M. , Mitani, G. , Kutsuna, T. , Nagai, T. , … Mochida, J. (2012). Cartilage repair in transplanted scaffold‐free chondrocyte sheets using a minipig model. Biomaterials, 33, 3846–3851. 10.1016/j.biomaterials.2012.01.056 22369960

[term3101-bib-0008] Hamahashi, K. , Sato, M. , Yamato, M. , Kokubo, M. , Mitani, G. , Ito, S. , … Mochida, J. (2015). Studies of the humoral factors produced by layered chondrocyte sheets. Journal of Tissue Engineering and Regenerative Medicine, 9, 24–30. 10.1002/term.1610 23165985

[term3101-bib-0009] Hangody, L. , Ráthonyi, G. K. , Duska, Z. , Vásárhelyi, G. , Füles, P. , & Módis, L. (2004). Autologous osteochondral mosaicplasty. Surgical technique. The Journal of Bone and Joint Surgery. American Volume, 86(A Suppl 1), 65–72. 14996923

[term3101-bib-0010] Ito, S. , Sato, M. , Yamato, M. , Mitani, G. , Kutsuna, T. , Nagai, T. , … Mochida, J. (2012). Repair of articular cartilage defect with layered chondrocyte sheets and cultured synovial cells. Biomaterials, 33, 5278–5286. 10.1016/j.biomaterials.2012.03.073 22551484

[term3101-bib-0011] Itokazu, M. , Wakitani, S. , Mera, H. , Tamamura, Y. , Sato, Y. , Takagi, M. , & Nakamura, H. (2016). Transplantation of scaffold‐free cartilage‐like cell‐sheets made from human bone marrow mesenchymal stem cells for cartilage repair: A preclinical study. Cartilage, 7, 361–372. 10.1177/1947603515627342 27688844 PMC5029565

[term3101-bib-0012] Kaneshiro, N. , Sato, M. , Ishihara, M. , Mitani, G. , Sakai, H. , Kikuchi, T. , & Mochida, J. (2007). Cultured articular chondrocytes sheets for partial thickness cartilage defects utilizing temperature‐responsive culture dishes. European Cells & Materials, 13, 87–92. 10.22203/ecm.v013a09 17516420

[term3101-bib-0013] Kaneshiro, N. , Sato, M. , Ishihara, M. , Mitani, G. , Sakai, H. , & Mochida, J. (2006). Bioengineered chondrocyte sheets may be potentially useful for the treatment of partial thickness defects of articular cartilage. Biochemical and Biophysical Research Communications, 349, 723–731. 10.1016/j.bbrc.2006.08.096 16949051

[term3101-bib-0014] Kokubo, M. , Sato, M. , Yamato, M. , Mitani, G. , Kutsuna, T. , Ebihara, G. , … Mochida, J. (2016). Characterization of chondrocyte sheets prepared using a co‐culture method with temperature‐responsive culture inserts. Journal of Tissue Engineering and Regenerative Medicine, 10, 486–495. 10.1002/term.1764 23868865

[term3101-bib-0015] Kwon, H. , Brown, W. E. , Lee, C. A. , Wang, D. , Paschos, N. , Hu, J. C. , & Athanasiou, K. A. (2019). Surgical and tissue engineering strategies for articular cartilage and meniscus repair. Nature Reviews. Rheumatology, 15(9), 550–570. 10.1038/s41584-019-0255-1 31296933 PMC7192556

[term3101-bib-0016] Maehara, M. , Sato, M. , Toyoda, E. , Takahashi, T. , Okada, E. , Kotoku, T. , & Watanabe, M. (2017). Characterization of polydactyly‐derived chondrocyte sheets versus adult chondrocyte sheets for articular cartilage repair. Inflammation and Regeneration, 37, 22 10.1186/s41232-017-0053-6 29259721 PMC5725814

[term3101-bib-0017] Mainil‐Varlet, P. , Aigner, T. , Brittberg, M. , Bullough, P. , Hollander, A. , Hunziker, E. , … International Cartilage Repair Society . (2003). Histological assessment of cartilage repair: A report by the Histology Endpoint Committee of the International Cartilage Repair Society (ICRS). The Journal of Bone and Joint Surgery. American Volume, 85(A Suppl 2), 45–57. 12721345

[term3101-bib-0018] Makris, E. A. , Gomoll, A. H. , Malizos, K. N. , Hu, J. C. , & Athanasiou, K. A. (2015). Repair and tissue engineering techniques for articular cartilage. Nature Reviews Rheumatology, 11(1), 21–34. 10.1038/nrrheum.2014.157 25247412 PMC4629810

[term3101-bib-0019] Mihara, M. , Higo, S. , Uchiyama, Y. , Tanabe, K. , & Saito, K. (2007). Different effects of high molecular weight sodium hyaluronate and NSAID on the progression of the cartilage degeneration in rabbit OA model. Osteoarthritis and Cartilage, 15, 543–549. 10.1016/j.joca.2006.11.001 17174116

[term3101-bib-0020] Mitani, G. , Sato, M. , Lee, J. I. , Kaneshiro, N. , Ishihara, M. , Ota, N. , … Mochida, J. (2009). The properties of bioengineered chondrocyte sheets for cartilage regeneration. BMC Biotechnology, 9, 17 10.1186/1472-6750-9-17 19267909 PMC2662823

[term3101-bib-0021] O'Driscoll, S. W. , Keeley, F. W. , & Salter, R. B. (1988). Durability of regenerated articular cartilage produced by free autogenous periosteal grafts in major full‐thickness defects in joint surfaces under the influence of continuous passive motion. A follow‐up report at one year. The Journal of Bone and Joint Surgery. American Volume, 70, 595–606. 3356727

[term3101-bib-0022] Saris, D. B. F. , Vanlauwe, J. , Victor, J. , Almqvist, K. F. , Verdonk, R. , Bellemans, J. , & Luyten, F. P. (2009). Treatment of symptomatic cartilage defects of the knee: characterized chondrocyte implantation results in better clinical outcome at 36 months in a randomized trial compared to microfracture. The American Journal of Sports Medicine, 37(suppl 1), 10–19. 10.1177/0363546509350694 19846694

[term3101-bib-0023] Sato, M. , Yamato, M. , Mitani, G. , Takagaki, T. , Hamahashi, K. , Nakamura, Y. , … Watanabe, M. (2019). Combined surgery and chondrocyte cell‐sheet transplantation improves clinical and structural outcomes in knee osteoarthritis. NPJ Regenerative Medicine, 4, 4–11. 10.1038/s41536-019-0069-4 30820353 PMC6384900

[term3101-bib-0024] Shimizu, R. , Kamei, N. , Adachi, N. , Hamanishi, M. , Kamei, G. , Mahmoud, E. E. , … Ochi, M. (2015). Repair mechanism of osteochondral defect promoted by bioengineered chondrocyte sheet. Tissue Engineering. Part A, 21, 1131–1141. 10.1089/ten.TEA.2014.0310 25396711 PMC4356224

[term3101-bib-0025] Steadman, J. R. , Rodkey, W. G. , & Briggs, K. K. (2002). Microfracture to treat full‐thickness chondral defects: Surgical technique, rehabilitation, and outcomes. The Journal of Knee Surgery, 15, 170–176. 12152979

[term3101-bib-0026] Takaku, Y. , Murai, K. , Ukai, T. , Ito, S. , Kokubo, M. , Satoh, M. , … Sato, M. (2014). In vivo cell tracking by bioluminescence imaging after transplantation of bioengineered cell sheets to the knee joint. Biomaterials, 35, 2199–2206. 10.1016/j.biomaterials.2013.11.071 24360579

[term3101-bib-0027] Takahashi, T. , Sato, M. , Toyoda, E. , Maehara, M. , Takizawa, D. , Maruki, H. , … Watanabe, M. (2018). Rabbit xenogeneic transplantation model for evaluating human chondrocyte sheets used in articular cartilage repair. Journal of Tissue Engineering and Regenerative Medicine, 12, 2067–2076. 10.1002/term.2741 30058138 PMC6221121

[term3101-bib-0028] Takatori, N. , Sato, M. , Toyoda, E. , Takahashi, T. , Okada, E. , Maehara, M. , & Watanabe, M. (2018). Cartilage repair and inhibition of the progression of cartilage degeneration after transplantation of allogeneic chondrocyte sheets in a nontraumatic early arthritis model. Regenerative Therapy, 9, 24–31. 10.1016/j.reth.2018.07.003 30525072 PMC6222284

[term3101-bib-0029] Tani, Y. , Sato, M. , Maehara, M. , Nagashima, H. , Yokoyama, M. , Yokoyama, M. , … Mochida, J. (2017). The effects of using vitrified chondrocyte sheets on pain alleviation and articular cartilage repair. Journal of Tissue Engineering and Regenerative Medicine, 11, 3437–3444. 10.1002/term.2257 28198149

[term3101-bib-0030] Toyoda, E. , Sato, M. , Takahashi, T. , Maehara, M. , Okada, E. , Wasai, S. , … Watanabe, M. (2019). Transcriptomic and proteomic analyses reveal the potential mode of action of chondrocyte sheets in hyaline cartilage regeneration. International Journal of Molecular Sciences, 21(1), 149 10.3390/ijms21010149 PMC6981399 31878307

[term3101-bib-0031] Yamashita, A. , Morioka, M. , Yahara, Y. , Okada, M. , Kobayashi, T. , Kuriyama, S. , … Tsumaki, N. (2015). Generation of scaffoldless hyaline cartilaginous tissue from human iPSCs. Stem Cell Reports, 4, 404–418. 10.1016/j.stemcr.2015.01.016 25733017 PMC4375934

